# Axes of alienation: applying an intersectional lens on the social contract during the pandemic response to protect sexual and reproductive rights and health

**DOI:** 10.1186/s12939-020-01245-w

**Published:** 2020-07-31

**Authors:** Jashodhara Dasgupta, Marta Schaaf, Sana Qais Contractor, Amanda Banda, Marisa Viana, Oksana Kashyntseva, Ana Lorena Ruano

**Affiliations:** 1 SAHAYOG, F 25 Hauz Khas Enclave, New Delhi, 110016 India; 2 Independent Consultant, 357 Sixth Ave, Brooklyn, NY 11215 USA; 3 COPASAH Sexual and Reproductive Rights Hub, CHSJ, Basement of Young Women’s Hostel No 2, Avenue 21, G block, Saket, New Delhi, 110017 India; 4 Wemos, Amsterdam, Hallmark House, 54 Siemert Road, New Doornfontein, Johannesburg, Gauteng 2094 South Africa; 5 RESURJ, RESURJ, C/O The Praxis Project, 1900 Fruitvale Avenue, #3D, Oakland, CA 94601 USA; 6 Center for Harmonization of Human Rights of the Scientific Research Institute of IP of National Academy of Law Sciences of Ukraine, Каzymyra Маlevycha, 11, Kyiv, 03150 Ukraine; 7 Center for International Health, University of Bergen / Center for the Study of Equity and Governance in Health Systems (CEGSS), Guatemala, Center for International Health, Postboks 7804, NO-5020 Bergen, Norway

**Keywords:** Social contract; Intersectionality; Pandemic; SRHR/sexual and reproductive health and rights; Marginalized groups

## Abstract

While economic inequalities have been a key focus of attention through the COVID 19 pandemic, gendered relations of power at every level have undermined health rights of women, girls and gender diverse individuals. Sexual and reproductive health rights (SRHR) have always been sites of power contestations within families, societies, cultures, and politics; these struggles are exacerbated by economic, racial, religious, caste, citizenship status, and other social inequities, especially in times of crisis such as these. Policy responses to the COVID pandemic such as lockdown, quarantine, contact tracing and similar measures are premised on the existence of a social contract between the government and the people and among people, with the health sector playing a key role in preventive and curative care.

We propose the use of an intersectional lens to explore the impact of the COVID-19 pandemic on the social contract, drawing on our field experiences from different continents particularly as related to SRHR. Along with documenting the ways in which the pandemic hinders access to services, we note that it is essential to interrogate state-society relations in the context of vulnerable and marginalized groups, in order to understand implications for SRHR. Intersectional analysis takes on greater importance now than in non-pandemic times as the state exercises more police or other powers and deploys myriad ways of ‘othering’.

We conclude that an intersectional analysis should not limit itself to the cumulative disadvantages and injustices posed by the pandemic for specific social groups, but also examine the historical inequalities, structural drivers, and damaged social contract that underlie state-society relationships. At the same time, the pandemic has questioned the status quo and in doing so it has provided opportunities for disruption; for re-imagining a social contract that reaches across sectors, and builds community resilience and solidarities while upholding human rights and gender justice. This must find place in future organizing and advocacy around SRHR.

The COVID19 pandemic has exposed many fault-lines across the world, among which unequal power relations have shown up most starkly [1]. While economic inequalities have been a key focus of attention, gendered relations of power at every level have undermined health rights of women, girls and gender diverse individuals. Sexual and reproductive health and rights (SRHR) have always been sites of power contestations within families, societies, cultures, and politics; these struggles are exacerbated by economic, racial, religious, caste, citizenship status, and other social inequities, especially in times of crisis [2]. In keeping with this recognition that axes of inequality influence who gets exposed, who gets sick, and who gets good health care, in this Commentary, we propose the use of an intersectional lens to explore the impact of the COVID-19 pandemic on the social contract, particularly as related to SRHR. Developed by African-American legal scholar Kimberle Crenshaw, ‘intersectionality’ is a lens that examines how intersecting identities, such as race, gender, class, and ability shape people’s experiences with state organs and society at large [3]. As a group of SRHR and equity researchers, activists, and professionals, we provide examples from the diverse countries in which we live and work, discuss how the pandemic hinders access to services, interrogate state-society relations in the context of vulnerable and marginalized groups, and describe the implications for SRHR.

Around the world, as a way to slow down the spread of COVID-19, countries have asked their populations to surrender some of their most cherished rights, including freedom of movement, association, and economic activity, all in the interest of the greater good. These measures are standard public health tools designed to keep populations safe and to keep the health system from collapsing. However, people voluntarily give up rights on the assumption that the government has the intent and capacity to act in the people’s best interest, and that it will continue to take steps to protect, respect, and fulfil basic human rights, including SRHR. Lockdown, quarantine, contact tracing and similar measures are premised on the existence of a social contract between the government and the people, and among people. This social contract confers obligations on all organs of the state, with the health sector playing a key role in preventive and curative care, while other sectors address the social determinants of risk and distribution of SRHR status and COVID-19.

However, this social contract was already wearing thin in many countries even before COVID-19. For ideological and political reasons, many governments and others with power were opposed to reproductive and sexual rights in general; while some were particularly concerned with suppressing these rights for specific populations, such as ethnic minorities or LGBTI individuals [4, 5]. Governments excluded crucial services such as abortion, contraception for adolescents, and outreach programs to meet the needs of marginalized groups from national policies and plans. They refused to address or even reinforced family and social dynamics that contributed to Gender-Based Violence, such as marital rape and intimate partner violence [6]. Moreover, neoliberal approaches to health services, such as austerity measures, privatization and generalized resource scarcity have for decades undercut government ability to provide health and other services that respond to people’s needs [7]. This has resulted in severe shortages and maldistribution of the health workforce and inequitable access to medicines, commodities and equipment while eroding robust engagement with communities. Unsurprisingly, the Lancet-Guttmacher 2018 Report concluded that almost all 4.3 billion people of reproductive age worldwide would have inadequate sexual and reproductive health services over the course of their lives [8].

The COVID-19 pandemic further exposes gaps in public health response, revealing how health systems lacked the resilience to weather the shock without significant losses in SRHR services. The impact that COVID-19 is having on SRH services and their availability, accessibility, and quality has been widely discussed in the context of the health sector [2, 9–12]. Logistical drivers, such as unstable supply chain and stockouts of commodities, limitations on movement and added burdens on the health workforce and health facilities mean people may not obtain the critical services they need, potentially impacting maternal morbidity and mortality, among other SRHR health outcomes [13, 14]. For example, even though the Brazilian Ministry of Health recognizes pregnant and post-partum women as at risk, it remains challenging to prevent pregnancy through access to contraceptives methods and procedures through the Unified Health System. The bulk of the health workforce in India for instance are the under-paid and under-equipped women frontline workers who are absorbed in handling surveillance and care [15]; for pregnant women the only recourse may just be the community birth attendants [16], who have long been delegitimized by the state. These disruptions also have political drivers, such as state governments in the United States and the Government of Ukraine determining that abortion, while legal, is not essential and thus prohibited during periods of lockdown [14, 17–19].

In addition to these well-documented problems, access has been disrupted to sexual and reproductive services and commodities provided outside of the health sector, such as through markets, schools, and drop-in centres. For example, emergency contraception and condoms are no longer available for adolescents and younger women, as many clinics and school outlets are shut in Sub-Sharan Africa and Latin America, even though at this time many are exposed to rape and sexual coercion within homes [20]. In Ukraine, La Strada, an organization providing domestic violence support, reported a 40% increase in complaints during the lockdown period. Beyond contraceptives, schools also provided meals for students, and menstrual sanitary products: crucial services that have now been suspended in India, South Africa, some parts of the United States, and beyond [21]. The closure of state borders even prevented biological parents from access to children born via surrogacy, as happened in the case of 46 births in Ukraine for example [22].

The state response to COVID-19 further eroded the social contract for gender diverse populations; for example, Peru, Colombia, and Panama have instituted gender-based quarantine measures challenging for those who may not fit into binaries, such as distinct days for men and for women to go out; this can cause increased harassment, discrimination, and incarceration of transgender and gender non-conforming individuals [23]. There was an observed increase in the number of feminicides and incidence of gender-based violence targeting the LGBTQ community throughout Latin America. The pandemic has fueled transphobia, and transgender persons in India report increased violence within families where they are trapped. They are unable to access HIV treatment or hormone therapy, or even get condoms through the support centres which have been forced to close.

Poorly regulated, and in some cases, predatory, for profit private health services may be the only option for women and families that have no access to public services that are overwhelmed by the pandemic. Decades of government failure to adequately regulate these providers has enabled the flourishing of private systems that offer steeply priced services that evade accountability for the quality of care they provide. With no other options for crucial SRH services such as emergency obstetric care, abortions, and long-acting reversible contraceptives, many women and girls utilize expensive, poor quality services that may push them further into poverty and even cause physical harm. Their ability to pay has been severely eroded by the economic crisis precipitated by the pandemic; women working in the informal sector generally have little access to social protection, health insurance, or other social welfare systems [24].

Women, girls and marginalized groups seeking SRHR have historically faced pervasive and systemic discrimination within many countries, delineating axes of alienation within communities and between communities and state actors. In a crisis, these proclivities are not dampened; on the contrary, the crisis provides new opportunities to deny care and stigmatize certain categories of people, blaming them for the problems encountered. An intersectional analysis would therefore be not just one that addresses the cumulative disadvantages and injustices posed by the pandemic for specific social groups, but also the structural drivers, historical inequalities, and damaged social contract that mediates this relationship.

Intersectional analysis takes on greater importance now than in non-pandemic times as the state exercises more police and other power: states can deploy myriad ways of ‘othering’ while dodging questions of governance and accountability. What we are seeing through the Covid-19 prism is the inability of the state to deliver its end of the bargain, promised by a social contract that has for years been stripped of its social and moral essence in providing for its people.

Those affected by unequal power relations continue to face discrimination and denial of care, while the avenues and possibilities for seeking redress or accountability are obscured. However, the visibility of the state response to COVID-19 also exposes the status quo and provides opportunities for disruption in economic, social, political and financing domains. An example is the ‘feminist economic recovery plan’ that calls for ‘centering the needs and experiences of native Hawaiian and immigrant women’ [25]. The opportunity presented through these challenges is in the re-imagination of a social contract that reaches across sectors, and builds community resilience and solidarities while upholding human rights and an intersectional approach to gender justice.

The implication for SRHR research and advocacy work is that it cannot merely be restricted to analyzing access and easing it through ensuring service guarantees, legislation, regulation, and supplies, although these are critical. It would imply transnational solidarity, support to existing movements of historically disadvantaged groups and community engagement in policy-making through expanded civic space for rights claiming, fostering commitment for inclusive, transparent and resilient health systems. An intersectional approach would include reversing austerity, creating a global health response based on solidarity and collaboration; building an equitable financial order that enables governments to invest in robust public goods, funding comprehensive social protection and universal health coverage packages, including all SRHR entitlements, with effective regulation of the private sector [26].

We need to recognize that it is not just the COVID-19 pandemic and its responses alone that are hindering access, but the long-standing damage that has been done to social (and gender) relationships, this new all-powerful unaccountable avatar of the state and forms of surveillance and discrimination internalized within communities. The canvas of enquiry and campaigning has to be much broader than it is at the moment; it must simultaneously recognize the multiple axes of alienation and seek to reimagine state-society relations. The fulfilment of SRHR will depend on the extent to which we can repair and resist further damage that is being done through social isolation, impaired civil society action and enhanced state authority.

## Data Availability

Not applicable.
